# Predominance of Viable Spore-Forming Piezophilic Bacteria in High-Pressure Enrichment Cultures from ~1.5 to 2.4 km-Deep Coal-Bearing Sediments below the Ocean Floor

**DOI:** 10.3389/fmicb.2017.00137

**Published:** 2017-02-06

**Authors:** Jiasong Fang, Chiaki Kato, Gabriella M. Runko, Yuichi Nogi, Tomoyuki Hori, Jiangtao Li, Yuki Morono, Fumio Inagaki

**Affiliations:** ^1^Hadal Science and Technology Research Center, Shanghai Ocean UniversityShanghai, China; ^2^Department of Natural Sciences, Hawaii Pacific University, HonoluluHI, USA; ^3^Department of Marine Biodiversity Research, Japan Agency for Marine-Earth Science and TechnologyYokosuka, Japan; ^4^Environmental Management Research Institute, National Institute of Advanced Industrial Science and TechnologyIbaraki, Japan; ^5^State Key Laboratory of Marine Geology, Tongji UniversityShanghai, China; ^6^Kochi Institute for Core Sample Research, Japan Agency for Marine-Earth Science and TechnologyKochi, Japan; ^7^Research and Development Center for Ocean Drilling Science, Japan Agency for Marine-Earth Science and TechnologyYokohama, Japan; ^8^Research and Development Center for Submarine Resources, Japan Agency for Marine-Earth Science and TechnologyYokosuka, Japan

**Keywords:** deep biosphere, endospore, piezophiles, high pressure, coal beds, marine sediment

## Abstract

Phylogenetically diverse microorganisms have been observed in marine subsurface sediments down to ~2.5 km below the seafloor (kmbsf). However, very little is known about the pressure-adapted and/or pressure-loving microorganisms, the so called piezophiles, in the deep subseafloor biosphere, despite that pressure directly affects microbial physiology, metabolism, and biogeochemical processes of carbon and other elements *in situ*. In this study, we studied taxonomic compositions of microbial communities in high-pressure incubated sediment, obtained during the Integrated Ocean Drilling Program (IODP) Expedition 337 off the Shimokita Peninsula, Japan. Analysis of 16S rRNA gene-tagged sequences showed that members of spore-forming bacteria within Firmicutes and Actinobacteria were predominantly detected in all enrichment cultures from ~1.5 to 2.4 km-deep sediment samples, followed by members of Proteobacteria, Acidobacteria, and Bacteroidetes according to the sequence frequency. To further study the physiology of the deep subseafloor sedimentary piezophilic bacteria, we isolated and characterized two bacterial strains, 19R1-5 and 29R7-12, from 1.9 and 2.4 km-deep sediment samples, respectively. The isolates were both low G+C content, gram-positive, endospore-forming and facultative anaerobic piezophilic bacteria, closely related to *Virgibacillus pantothenticus* and *Bacillus subtilis* within the phylum Firmicutes, respectively. The optimal pressure and temperature conditions for growth were 20 MPa and 42°C for strain 19R1-5, and 10 MPa and 43°C for strain 29R7-12. Bacterial (endo)spores were observed in both the enrichment and pure cultures examined, suggesting that these piezophilic members were derived from microbial communities buried in the ~20 million-year-old coal-bearing sediments after the long-term survival as spores and that the deep biosphere may host more abundant gram-positive spore-forming bacteria and their spores than hitherto recognized.

## Introduction

The deep subseafloor biosphere is unique in that microorganisms long persist in the high-pressure environment over geologic time. Pressure is one of the important physical properties of subseafloor sedimentary habitats, which most likely affects microbial cell viability, growth, and physiology (Allen et al., 1999; Fang et al., 2004; Fichtel et al., 2015). Pressure ranges over four-orders of magnitude in the surface biosphere (Bartlett et al., 1995) whereas probably more in the deep subseafloor biosphere. In such habitats under the deep-sea bed, there may be microorganisms that require high-pressure conditions for optimal growth, so called “piezophiles” (Yayanos, 1995), whose activity possibly plays important roles in global biogeochemical cycles (e.g., Fichtel et al., 2015).

A better understanding of microbial metabolism of subsurface microorganisms is essential to help delineate their impact on biogeochemical cycling in the deep biosphere. However, we still do not know the metabolic activities of pressure-adapted subsurface microorganisms and the pressure (depth) and habitability limit of the deep biosphere, as the deep subsurface prokaryotic cells are mostly resistant to cultivation and only <0.1% of all microscopically and/or molecular genetically detected cells have been isolated and identified (Batzke et al., 2007; Sass and Parkes, 2011; Ciobanu et al., 2014; Russell et al., 2016). Thus far, studies of the deep subsurface biosphere have yielded relatively few pressure-adapted bacterial isolates for laboratory studies of their physiology, ecology and biogeochemical functions (Bale et al., 1997; Russell et al., 2016). The pressure (depth) and habitability limits of the deep subseafloor life remain poorly constrained.

In 2012, the Integrated Ocean Drilling Program (IODP) Expedition 337 provided an unprecedented opportunity to study piezophilic microbial communities, which may include some spore-formers buried in the deep and old coal-bearing sediment. Using the riser-drilling research vessel *Chikyu*, we drilled at Site C0020 in a former forearc basin in the north western Pacific off the Shimokita Peninsula, Japan (Inagaki et al., 2012, 2015; Gross et al., 2015; Glombitza et al., 2016; Tanikawa et al., 2016). The primary goal of this study was to investigate microbial communities of three sediment core samples from the depths of ~1.5 to 2.4 km below the ocean floor and, by isolating piezophilic bacterial strains, to study metabolism of the piezophilic isolates in the deep subseafloor biosphere. Our results show that enrichment cultures from the very deep sedimentary microbial communities were dominated by gram-positive endospore-forming piezophilic bacteria. The successful isolation and cultivation of these gram-positive piezophilic bacteria underline that microorganisms were revivable after the long-term burial as spores.

## Materials and Methods

### Sampling Site, Sedimentological and Geochemical Characteristics of the Sediment Samples

Deep subseafloor sediment samples used in this study were obtained by the riser-drilling technology of the deep-sea drilling research vessel *Chikyu* during the Integrated Ocean Drilling Program (IODP) Expedition 337 at Site C0020 (41°10.60′ N, 142°12.03′ E; water depth 1,180 m) off the Shimokita Peninsula, Japan (Supplementary Figure 1; Inagaki et al., 2012, 2015). Three sediment samples, 6R-3, 19R-1~25R-3, and 29R-7 were obtained from 1,498 mbsf, 1,951~1,999 mbsf, and 2,406 mbsf immediately after core recovery and the X-ray computed tomography (CT) scan. All sediment samples were blackish and associated with Miocene lignite coals that contain possibly biogenic pyrite veins (Gross et al., 2015; Glombitza et al., 2016).

Lithological and geochemical characteristics of the sediment samples were determined on the *Chikyu* by combined analysis of cuttings and cores (Inagaki et al., 2012, 2015; Gross et al., 2015; Glombitza et al., 2016; Tanikawa et al., 2016). Generally, two samples at greater depths, 19R-1~25R-3 and 29R-7, had rather different lithological and geochemical characteristics to the shallow sample 6R-3. Sample 19R-1~25R-3 represents deposition in a back-barrier shallow marine environment with wetlands in early middle Miocene (Gross et al., 2015). The sedimentary unit was characterized by sandstones, siltstones and coaly shale with excellent pollen and spore assemblages. Total organic carbon (TOC) content of the sediment was up to 30% (Inagaki et al., 2012). Sample 29R-7 was deposited in late Oligocene in an environment similar to that for 19R-1~25R-3. The lithology includes sandstone intercalated with silt and a thin coal layer. In contrast, the sediment unit represented by 6R-3, with massive sandstone and siltstone and marine fossiliferous material was deposited in an offshore marine environment. Thus, sediments of 19R-1~25R-3 and 29R-7 represent deposition in a transitional depositional environment with obvious terrestrial influence, whereas sample 6R-3 represents a cool-water continental shelf environment (Inagaki et al., 2012; Gross et al., 2015).

### High Pressure Enrichment and Isolation of Deep Subseafloor Piezophilic Bacteria

The three sediment samples (about 10 g each), 6R-3, 19R-1~25R-3, and 29R-7, were set up as three high-pressure (HP) enrichment cultures to be used for the downstream isolation of pure cultures, designated as 6R-3HP, 19R-1~25R-3HP, and 29R-7HP, according to the previously published procedure (see Kato, 2011). During Expedition 337 and thereafter, the three HP enrichment cultures were kept under the pressure *in situ* (i.e., 35 MPa), anoxic, and the respective environmental temperatures at 35, 45, and 55°C, respectively. The HP cultivation was conducted using Marine Broth 2216 (MB 2216, Difco) liquid medium for 3 months. The HP enrichment cultures were then transferred to new MB 2216 (1% seed, v/v) and the procedure was repeated under the same temperature and pressure condition for three times. Finally, the cultures were plated on MB 2216 agar medium (1.2%, w/v) for single colony isolation at atmospheric condition. Further growth experiments of bacterial isolates were performed under anoxic conditions using the AnaeroPack chamber (Mitsubishi Gas Chemical, Tokyo) at the same conditions described above.

### Taxonomic Analysis of HP Enrichment Cultures Based on 16S rRNA Gene Sequences

**FIGURE 1:**
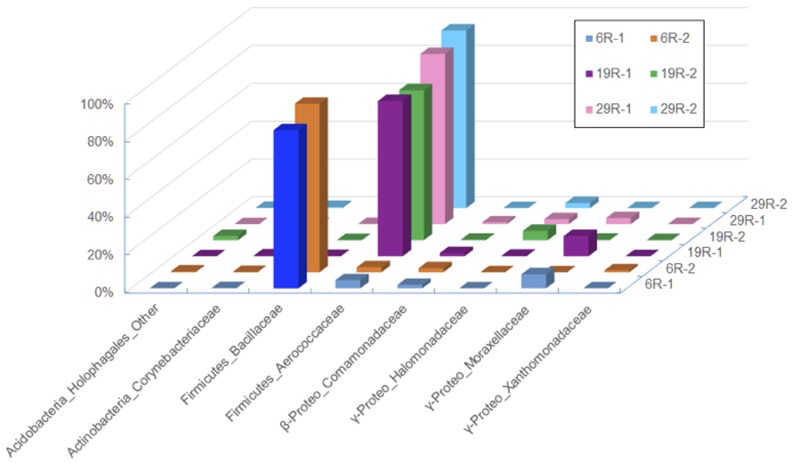
**Bacterial compositions showing the relative abundance of family level classification among different sediments and aqueous phases after the first high-pressure enrichment cultivation.** Only bacterial lineages whose abundances are >1% of reads are shown.

To study taxonomic composition and community structure of HP enrichment culture samples, we extracted DNA from each sediment and aqueous phase of the first enrichment slurry: for the sediment, we designated the sample name as 6R-1 for Core 6R-3, 19R-1 for Core 19R-1~25R-3, and 29R-1 for Core 29R-7; for the medium aqueous phase, 6R-2 for Core 6R-3, 19R-2 for Core 19R-1~25R-3, and 29R-2 for Core 29R-7 (**Figure 1**).

Total DNA was extracted from sediment and aqueous phases in the first HP enrichment cultures using a Power soil DNA extraction kit (MoBio Laboratories, Carlsbad, CA, USA), according to the manufacturer’s protocol. The 16S rRNA gene amplicon sequencing was performed to analyze taxonomic composition of three HP enrichment cultures using MiSeq system (Illumina, USA) as described previously (Aoyagi et al., 2015). Briefly, the primer set used for the amplicon sequencing was 515f/806r, which primer sequences were both modified to include an Illumina adapter region, and the reverse primer was encoded with 12-bp barcodes (Caporaso et al., 2012). PCR condition was as described previously (Navarro et al., 2015). The PCR amplicons were purified, first with an AMPure XP Kit (Beckman Coulter, USA), and the second with a QIAquick gel extraction kit (QIAGEN, USA). The concentration of the purified amplicon DNA was determined with a Quant-iT PicoGreen dsDNA reagent and kit (Life Technologies, USA). An appropriate amount of the purified amplicon and an internal control (PhiX Control V3; Illumina) were subjected to sequencing with a 300-cycles MiSeq Reagent kit (Illumina) and a MiSeq sequencer. The removal of the PhiX, low-quality (Q < 30) and chimeric sequences, and the assembly of the paired-end sequences were discarded as described previously (Itoh et al., 2014). The sequences in the sequence libraries were characterized phylogenetically by using the QIIME software (Caporaso et al., 2010). Alpha-diversity indices (e.g., Chao1, Shannon, and Simpson reciprocal) and the weighted UniFrac distances for principal coordinate analysis (PCoA) were calculated with the QIIME software (Caporaso et al., 2010) on the base of sequences obtained as above.

### Characterization of Piezophilic Bacterial Isolates

DNA of piezophilic bacterial isolates was purified using the method of Saito and Miura (1963), and 16S rRNA genes were amplified by PCR using the primers Eubac27F and Eubac1492R (DeLong, 1992). Amplified sequences (~1.5 kb) were determined using a Perkin-Elmer 3730xl DNA sequencer (Perkin-Elmer/Applied Biosystems, USA). PCR amplification was performed with a DNA Thermal Cycler model 9600 (Perkin-Elmer/Cetus, Co.) by using 50 ul of the PCR reaction mixture under the conditions recommended by the manufacturers (Takara, Co.) (Kato et al., 1995). A total of 30 cycles of amplification were performed with temperate DNA denaturation at 95°C for 1.5 min, primer annealing at 55°C for 1.5 min, and primer extension at 72°C for 1.5 min (DeLong, 1992). A phylogenetic tree was constructed based on 16S rRNA gene sequences. Nucleotide substitution rates (K_nuc_: Kimura, 1980) were determined, and the distance matrix tree was constructed by the neighbor-joining method (Saitou and Nei, 1987) using the Clustal X 2.0 program (Larkin et al., 2007). Cell morphology was observed under scanning electron microscope (SEM; model JSM-6700F, JEOL, Co., Japan) and transmission electron microscope (TEM; model JEM-1210, JEOL, Co., Japan) of the negative stained cells (Nogi et al., 1998) (Supplementary Figure 2). Phenotypic and biochemical characterizations were completed using the API50CHB strips (BioMerieux, Lyon), according to the manufacturer’s instructions. The G+C content (mol%) was measured by reversed-phase high-performance liquid chromatography (Tamaoka and Komagata, 1984). For analysis of relatedness between the isolates and reference species, DND-DNA hybridization was performed at 40°C for 3 h and measured fluorometrically according to the method of Ezaki et al. (1989). The isolated strains were deposited to the Japan Collection of Microorganisms (JCM, Tsukuba-City, Japan), and the strain numbers were JCM31625 (6R3-1), JCM31626 (6R3-15), JCM31627 (19R1-5), and JCM31625 (29R7-12).

### Fatty Acids Analyses

Total lipids were extracted from microbial cells using a one-phase solvent system (Fang and Findlay, 1996). The total lipids obtained were separated into neutral lipids, glycolipids, and phospholipids using miniature champagne columns (Supelco, Inc., Bellefonte, PA, USA) (Fang et al., 2007). The phospholipid fraction was subjected to a mild alkaline trans-methylation procedure to produce fatty acid methyl esters (FAMEs). FAMEs were analyzed on an Agilent 6890 GC interfaced with an Agilent 5973N Mass Selective Detector. Individual fatty acids were identified according to their mass spectra.

### Growth Experiments

High-pressure cultivation tests of the isolates were performed at various pressures of 0.1–70 MPa with an increment of 10 MPa, according to the procedure described by Kato et al. (1995). Optimal temperature conditions were determined at the range of 10–60°C, and optimal salt conditions were determined at the concentration range of 0–11 ‰ NaCl, using a bio-photo recorder system (model TVS126MA, Advantec, Japan) on the basis of optical density at 600 nm (OD_600_).

### Nucleotide Sequence Accession Numbers

The 16S rRNA gene sequences of isolates reported in this paper were deposited in the DDBJ (DNA Data Bank of Japan) under accession numbers LC155963 – LC155966. The nucleotide sequences obtained from the Illumina sequencing of 16S rRNA genes were deposited in the MG-RAST database as “Isolation and characterization of piezophilic microorganisms from 1.5 and 2.4 km-deep subseafloor sediment core samples” project under accession ID 4695872.3-4695877.3.

## Results and Discussion

### Taxonomic Composition of High-Pressure Enrichment Communities

Taxonomic classification analysis of HP enrichment cultures clearly indicated that cultivated piezophilic communities were dominated by members of the phylum Firmicutes with >80% of the reads, followed by members of Gamma-proteobacteria, which sequence frequency ranged between 1.2 and 11.6% (**Figure 1**). No obvious differences in bacterial community compositions between the sediment and aqueous phases were observed after the first HP cultivation (**Figure 1**), suggesting that no significant microbial fractionation occurred between the sediment (sedimentary microbes) and aqueous phases (free-living microbes) during HP cultivation. Thereafter, we decided to use whole (i.e., mixed) microbial communities for the DNA analysis of HP enrichment cultures.

**FIGURE 2:**
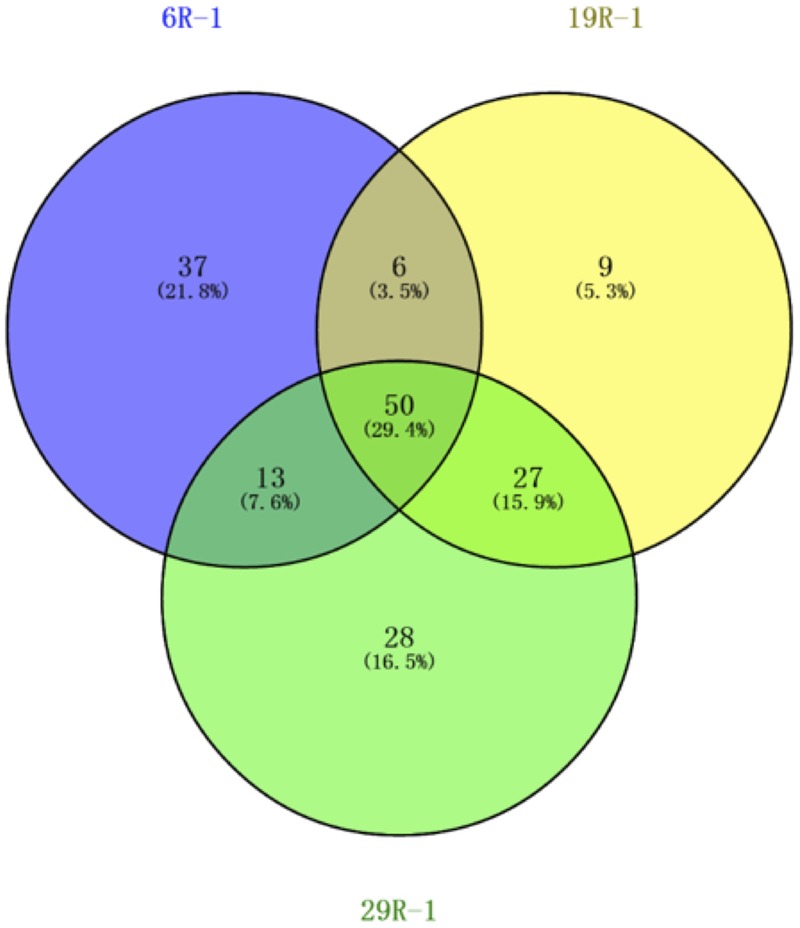
**Venn diagram of OTUs for bacterial diversity of the first high-pressure enrichment cultures.** Unique and shared OTUs between the sample pairs are based on 97% similarity.

There were 39,020, 9,899, and 44,365 qualified 16S rRNA gene sequences recovered from HP enrichment samples 6R-1, 19R-1, and 29R-1, respectively. The sequences represented 12 phyla, 39 classes, 47 orders, 91 families, and 129 genera. At the phylum level, the vast majority of the recovered sequences was found to be the phylum Firmicutes, which was 88.8, 83.8, and 91.2% of the total sequence read number for 6R-1, 19R-1, and 29R-1, respectively (Supplementary Figure 3). The next most abundant phyla were Proteobacteria and Actinobacteria. Based on relative abundance, *Bacilli* (88.7, 83.6, and 91.6%), Gamma-proteobacteria (7.7, 11.6, and 6.8%) and Beta-proteobacteria (2.2, 2.6, and 1.2%) were the most abundant classes in those enrichment samples (data not shown). However, there are significant differences in HP-cultivated bacterial communities between 6R-3 and two deeper samples (19R-1~25R-3 and 29R-7), possibly corresponding to the lithological characteristics (6R-3 was deposited in an offshore marine environment whereas 19R-1~25R-3 and 29R-7 were deposited in a transitional to shallow marine environment) (**Figure 1**). At the order level, members of the *Bacillales* dominated enriched bacterial communities in 6R-1 (84.0%), whereas members of the *Lactobacillales* were predominant in 19R-1 (90.3%) and 27R-1 (82.5%) (Supplementary Figure 3). The difference in cultured bacterial communities between shallow and deep sediment samples could be further illustrated at the genus level. The most abundant genus in samples 19R-1 and 29R-1 was *Alkalibacterium* (81.0 and 88.8% of the total sequence reads, respectively). In contrast, the most abundant genus in sample 6R-1 was *Marinilactibacillus* (83.4%), which is a fermentative marine lactic acid bacterium that has been isolated from shallow subsurface sediments in the northwestern Pacific, being either psychrophilic (Ishikawa et al., 2003) or piezophilic (Toffin et al., 2005) (Supplementary Figure 3). However, given the geochemical conditions at Site CC0020, it is unlikely that the Shimokita coalbed sediments host psychro-piezophilic bacteria, but rather, meso- and thermo-piezophilic bacteria (see below). Based on the cut-off value of 97% similarity, a total of 170 OTUs were identified from three HP enrichment culture samples. The relatively low number of OTUs suggests generally low species richness in the cultivated microbial community. The Venn diagram showed that three sediment samples 6R-1, 19R-1 and 29R-1 shared 50 OTUs (29.4%), with each possessing 37 (21.8%), 9 (5.3%), and 28 (16.5%) unique OTUs, respectively (**Figure 2**). PCoA clearly separated HP-cultured communities in sediment 6R-1 from 19R-1 and 29R-1, with the first coordinate accounting for 70.2% and the second for 17.0% of the samples’ variance (Supplementary Figure 4).

It is instructive to note that microbial communities cultivated under the HP condition mainly consisted of gram-positive endospore-forming bacteria. Within the phylum Firmicutes, members of the families *Bacillaceae* and *Aerococcaceae* are typical low G+C content gram-positive lineages commonly present in terrestrial soil and sediment, whereas members of the phylum Actinobacteria, detected in these sediments with a range of 0.2~2.8%, are well-known high G+C content gram-positive bacteria in various marine and freshwater environments (e.g., Ventura et al., 2007). This result corroborates that of Inagaki et al. (2015), which showed that Firmicutes and Actinobacteria were detected as indigenous bacterial communities in deep coal-bearing sediments at Site C0020. In addition, we employed the same cultivation method that has been previously used for deep-sea piezophilic bacteria and resulted in successful isolation of both gram-negative and gram-positive piezophilic bacteria (e.g., Kato and Nogi, 2001). These results suggest that gram-positive spore-forming piezophilic bacteria probably constitute a large part of cultivable deep subseafloor bacterial communities at Site C0020.

### Isolation and Characterization of Piezophilic Bacteria

**FIGURE 3:**
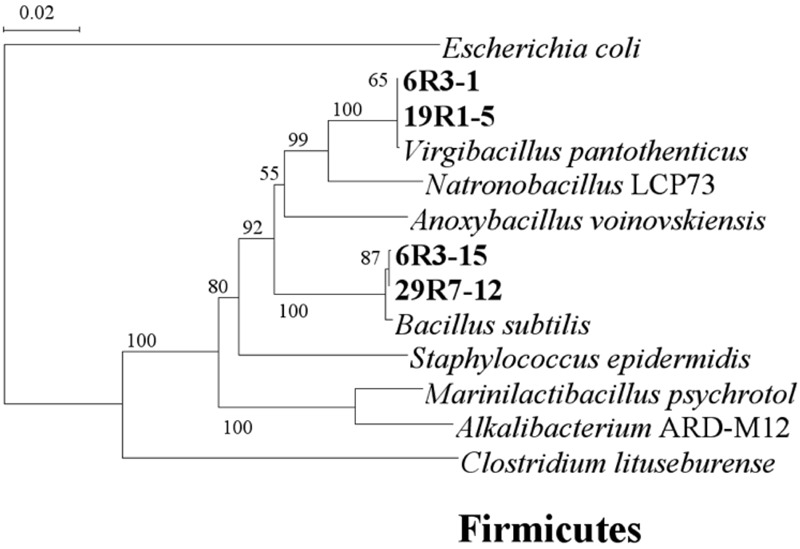
**Phylogenetic tree based on 16S rRNA gene sequences showing the relationship among the isolated subsurface bacteria within the phylum Firmicutes.** The scale represents the average numbers of nucleotide substitutions per site. Bootstrap values are shown for frequencies per 1,000 times.

To study more about physiology of piezophilic bacteria, we isolated four bacterial strains, named 6R3-1, 6R3-15, 19R1-5, and 29R7-12, from each HP enrichment culture of 6R-3 HP, 19R-1~25R-3 HP, and 29R-7 HP, respectively. Phylogenetic analysis of each16S rRNA gene sequence revealed that bacterial isolates 6R3-1 and 19R1-5 are closely related to *Virgibacillus pantothenticus* whereas isolates 6R3-15, 29R7-12 are to *Bacillus subtilis* (**Figure 3**). DNA–DNA hybridization comparing the isolated strains and reference species *V. pantothenticus* and *B. subtilis* suggests that strains 6R3-1 and 19R1-15 are the same species as *V. pantothenticus* (91.7–100% of homology), and strains 6R3-15 and 29R7-12 are the same as *B. subtilis* (70.4–90.7% of homology) (Supplementary Table 1). The physiological data further confirmed that isolate 6R3-15 is identical to 19R1-5, and 6R3-15 to 29R7-12 (Supplementary Table 1). Therefore, we selected the bacterial isolates 19R1-5 and 29R7-12 as representative piezophilic isolates for further characterizations.

**FIGURE 4:**
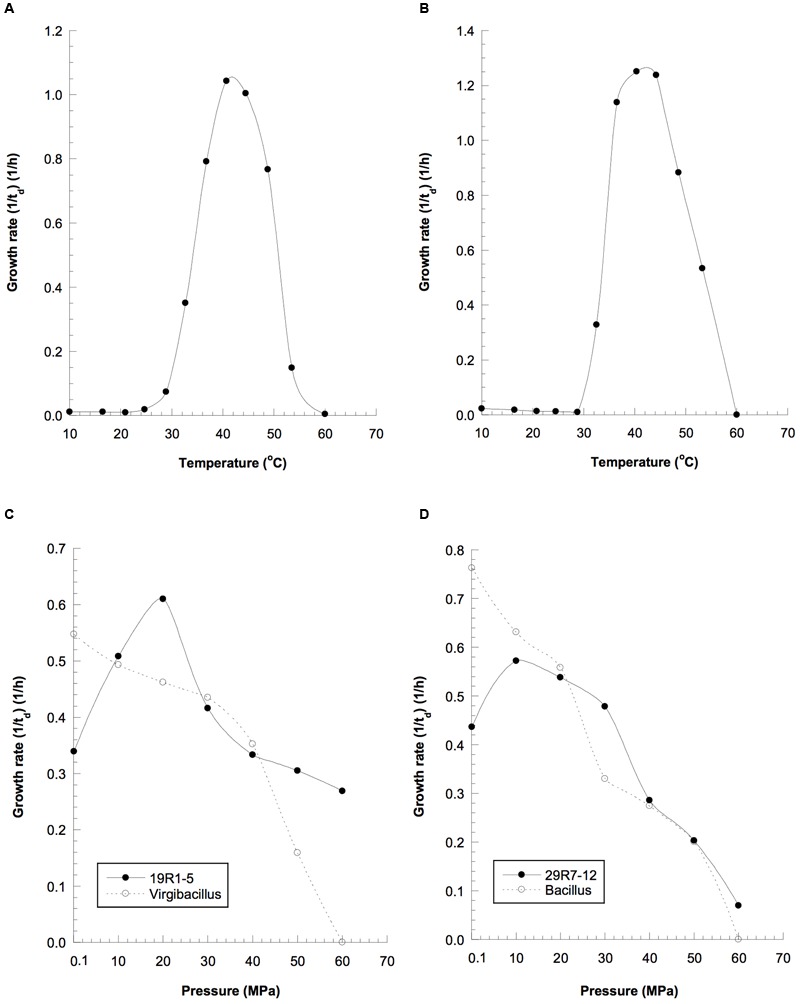
**Growth properties of the spore-forming piezophilic bacterial isolates and the closely related reference strains at elevated temperatures: (A)** strain 19R-1, **(B)** strain 29R-1; and hydrostatic pressures: **(C)** solid line, strain 19R1-5; dotted line, *Vergibacillus pantothenticus*; **(D)** solid line, strain 29R7-12; dotted line, *Bacillus subtilis*, t_d_, doubling time (in hours).

Isolates 19R1-5 and 29R7-12 were characterized as gram-positive facultative anaerobic bacteria, able to grow under both anoxic and oxic conditions. The strains were also able to grow not only at high pressures but also at relatively high temperature conditions (**Table 1**). Strains 19R1-5 and 29R7-12 exhibited optimal growth at 42 and 43°C, respectively, lower than *in situ* habitat temperatures of 46°C for 19R1-5 and 58°C for 29R7-12 (**Figures 4A,B**). The isolates also showed piezophilic growth with their optimum growth pressure of 20 and 10 MPa for 19R1-5 and 29R7-12, respectively (**Figures 4C,D**). It is apparent that the optimal growth pressures for the bacterial isolates are considerably lower than habitat pressures, which are 53 and 65 MPa (as hydrostatic pressure) for 19R1-5 and 29R7-12, respectively. The reason for this large pressure difference is possibly because those piezophilic isolates are derived from spores in coaly shale sediments, which have originally been buried from warm and shallow coastal environments (Gross et al., 2015; Inagaki et al., 2015). Alternatively, it has been observed that the upper cardinal temperature for piezophilic bacterial growth can be extended by high pressure, but the inverse tests have not been done. In our study, the high temperature and high pressure growth experiments were conducted independently; i.e., in determining optimal growth pressure of the isolates, pressure was varied while maintaining the culture at *in situ* temperature, whereas the growth temperature experiments were carried out at the atmospheric pressure condition, rather than at the *in situ* pressure. This experimental setup may explain why the optimal growth temperatures for two isolates were lower than the *in situ* habitat temperatures, but is in contrast to the observation of deep-sea piezophilic bacteria that showed more stenothermal growth for those from deeper depths of the original habitat (e.g., Yayanos, 1986).

**Table 1:** Growth characteristics of the bacterial isolates and reference species.

Features	19R1-5	*V. pantothenticus*	29R7-12	*B. subtilis*
Growth characteristics				
Optimal pressure (MPa)	20	0.1	10	0.1
Optimal temperature (°C)	42	37	43	37
Growth at NaCl (%)	1–8	NT	0–8	NT
Optimum NaCl (%)	2–4	NT	0–2	NT
Anaerobic growth	+	-	+	-
Oxidation of carbon sources				
Glycerol	+	+	-	+
d-glucose	+	+	-	+
d-fructose	+	+	-	+
l-rhamnose	+	-	+	+
d-sorbitol	+	+	-	+
Methyl-α,d-mannnopyranoside	-	-	-	+
Amygdalin	+	-	+	+
Arbutin	+	-	+	+
Salicin	+	-	+	+
d-melibiose	+	-	-	-
Starch	+	-	+	+
Glycogen	+	-	-	-
Gentibios	-	-	-	+
d-turanose	+	-	+	+
G+C content (mol%)	38.4	38.0	44.8	45.0
Motility	+	+	-	+

*NT, no test.*

The two bacterial isolates 19R1-5 and 29R7-12 exhibited many phenotypic characteristics common to the reference species *V. pantothenticus* and *B. subtilis*, such as the G+C content, composition of cellular fatty acids, and utilization of different carbon substrates (**Tables 1** and **2**). The two strains are facultative anaerobic chemoorganotrophs, displaying both respiratory and fermentative types of metabolism. *Iso*- and *anteiso*-branched fatty acids dominate the cellular lipids. The preponderance of methyl-branched fatty acids in both strains is in concordance with gram-positive Firmicutes. Taken together, these results indicate that bacterial isolates 19R1-5 and 29R7-12 are gram-positive spore-forming piezophilic bacteria (Fang et al., 2010), of the same species as *V. pantothenticus* and *B. subtilis*, respectively.

**Table 2:** Comparison of the fatty acid profile of the isolates and the related reference species as measured by GC analysis of their methyl esters.

Fatty acids	% of the total fatty acids			
	19R-5	*V. pantothenticus^∗^*	29R7-12	*B. subtilis^∗∗^*
*anteiso*-C_13:0_	–	0.5	-	
*iso*-C_14:0_	-	4.3	-	0.99
C_14:0_	-	1.2	-	
*iso*-C_15:0_	3.6	15.8	24.7	30.6
*anteiso*-C_15:0_	48.5	47.4	36.0	38.8
*iso*-C_16:0_	3.2	8.3	6.8	
C_16:0_	1.1	5.2	2.4	4.69
*iso*-C_17:0_	1.7	2.8	19.1	7.45
*anteiso*-C_17:0_	42.0	13.5	10.9	7.55

*Only fatty acids accounting for greater than 0.5% of the total fatty acid content are listed.*

*^∗^Data from Heyndrickx et al. (1998).*

*^∗∗^Data from Gatson et al. (2006).*

### Implications for Cultivable Piezophilic Bacteria in the Deep Subseafloor Biosphere

Interpreting the origin of cultivable piezophilic bacteria in deep and old subseafloor sediments is a particular challenge for the scientific ocean drilling community. Inagaki et al. (2015) and others suggested that the depositional environment and source of the stratified sediment exert a dominant control on indigenous microbial communities in the deep sedimentary biosphere. Site C0020 is in a former forearc basin where organic-rich terrestrial deposits are expected to be dominant and pervasive in the initial development stage of the basin (Gross et al., 2015). Subsequent subsidence of the basin allowed the deposition of marine sediments overlying the deep terrigenous coal-sand formation. Our results also showed that microbial communities in deep coalbed layers (samples 19R-1 and 29R-1) resemble those commonly found in terrestrial surface soil environment where phyla Firmicutes, Actinobacteria, Proteobacteria, Acidobacteria and Bacteroidetes (designated “FAPAB”) dominate. The Venn diagram for the assemblage of first HP-enrichment cultures clearly shows that sediment samples 19R-1 and 29R-1 from deeper depths shared more OTUs (16%) than each shared with 6R-1, 3.5 and 7.6%, from the shallower depth. We hypothesize that the deposition of terrestrial sediment and the FAPAB buried therein help the organisms persisting in the subsurface for prolonged periods, and the persisted FAPAB species provides a seed bank for populating the deep-biosphere community under the increasing selection pressure with the burial depth and time. As sediment deposition and progressive burial of sediments continues in the basin, temperature and pressure of the buried sediment also increase, which may selectively increase the relative population of cultivable meso- and thermo-pizophilic bacteria in the deep sedimentary biosphere. We further deduce that microbial communities in deeper sedimentary layers may be more or less inherited from the initially buried sediment microbial communities and that the current rare microbial taxa in the rare biosphere may be originated from the former common biosphere and may have served as a seed bank to deeper microbial ecosystems that adapt to geophysical and geochemical changes.

**FIGURE 5:**
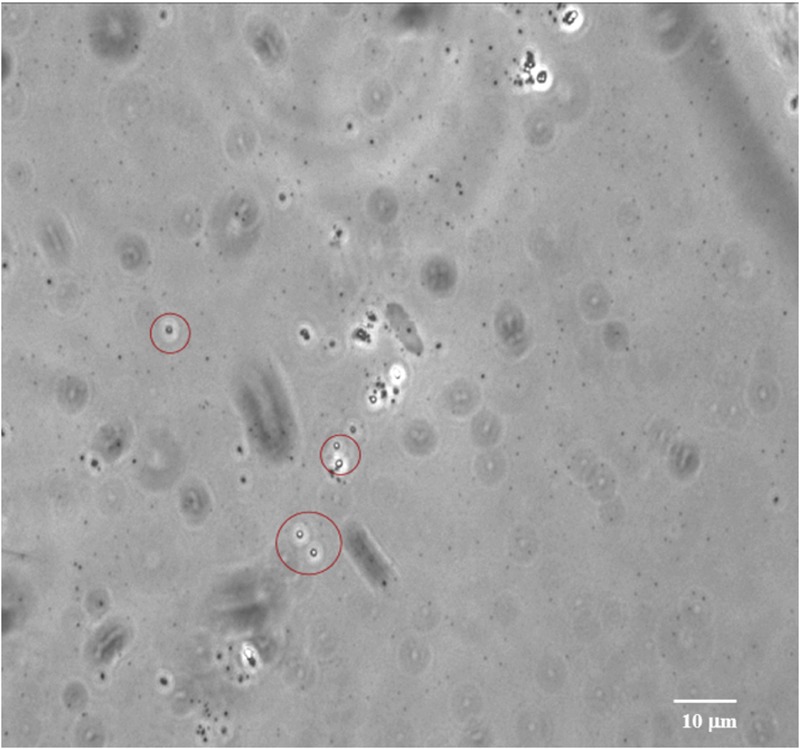
**Bacterial endospores observed in the subsurface sediment at 2406 mbsf at Site C0020.** Image was taken using an optical microscope (Olympus Co., Tokyo, Japan).

Bacterial sporulation in the natural environment is one of the self-preservation processes that allow bacteria, under unfavorable conditions, to preserve and propagate the genetic information and functional survivability. Endospores are the most resistant cellular structure (Abecasis et al., 2013; Filippidou et al., 2016) and can remain viable for 1000s (Gest and Mandelstam, 1987) or even millions of years (Vreeland et al., 2000). In a previous study, spores of *V. pantothentics* and *B. marismortui* were isolated from a 250-million-year-old salt crystal (Vreeland et al., 2000). Recent studies showed that bacterial endospores are globally distributed in shallow to deep subseafloor sediments (Müller et al., 2014), as abundant as vegetative cells (Langerhuus et al., 2012; Lomstein et al., 2012), and generally increase with depth (Batzke et al., 2007; Fichtel et al., 2008). It is likely that the formation of spores and other quiescent cell forms allows bacteria to persist over geologic time, and perhaps more importantly, spores may contribute as a seed bank to the ecosystem development in the deep biosphere, since they are highly resistant and less prone to viral attack or predation (Sogin et al., 2006; Pedrós-Alió, 2007; Hubert et al., 2010). Consequently, they can be expected to accumulate in marine sediments and may contribute greatly to the total biomass of deep subseafloor microbial communities (Lomstein et al., 2012). Our bacterial isolates are gram-positive endospore-forming piezophilic bacteria. Many spores have been observed in the HP enrichment cultures and also in the pure cultures of the bacterial isolates (**Figure 5**), which is in good agreement with a bioreactor cultivation experiment for ~2 km-deep methanogenic communities associated with lignite coals (Inagaki et al., 2015). The results in this study augment a ‘paleome’ concept, a pool of ancient DNA and/or descendants preserved in sediments buried millions of years, which can provide insights into ancient genetic records and/or microbial ecosystems (Inagaki et al., 2005, 2015; Glombitza et al., 2016). To verify these hypotheses, more intensive cultivation-dependent studies of spores in deep and hot sedimentary systems will be required because it was found to be difficult to obtain metagenomic information from the deeply buried spore pool (Kawai et al., 2015). This is one of our on-going efforts in the exploration of the deep subseafloor biosphere.

## Conclusion

Unlike the surface biosphere where gram-negative bacteria are predominant (e.g., Gontang et al., 2007), the deep subseafloor biosphere may host more abundant spore-forming piezophilic bacteria, as demonstrated in this study. Even as spores, the deeply buried quiescent community may have the capacity to impact their surrounding geochemical environment (Hubert et al., 2010), and play an integral role in microbial ecosystem-development as a seed pool in the deeper sedimentary biosphere. It is also clear from this study that bacteria can be readily isolated from the deep subseafloor when a special physiological group (e.g., piezophiles) is targeted and appropriate cultivation methods are applied. Our results underline that the deeply buried microorganisms are still alive and revivable. The continued use of cultivation-dependent approaches will lead to the discovery of additional pressure-adapted gram-positive bacteria and their spores, and provide a direct means to learn more about their adaptation and survival strategies as well as the evolution in the deep subseafloor biosphere.

## Author Contributions

JF and CK designed the research. TH, YM, and FI collected the core samples. CK, GR, and YN conducted cultivation experiments. TH and JL performed molecular and electron microscopic analyses. JF and CK wrote the manuscript with significant input from FI. All authors contributed to interpretation of data.

## Conflict of Interest Statement

The authors declare that the research was conducted in the absence of any commercial or financial relationships that could be construed as a potential conflict of interest.
