# Supplementary material for: Predominance of Viable Spore-Forming Piezophilic Bacteria in High-Pressure Enrichment Cultures from ~1.5 to 2.4 km-Deep Coal-Bearing Sediments below the Ocean Floor
Source: Front Microbiol. 2017 Feb 6;8:137. doi: 10.3389/fmicb.2017.00137 (PMC5292414; doi:10.3389/fmicb.2017.00137)
Supplement: Supplementary file 1 [file Data_Sheet_1.pdf]

Supplementary Material

Viable Spore-Forming Thermo-piezophilic Bacteria Dominate Microbial Communities in 1.5 to 2.4 km-Deep Subseafloor Sediment of the Northwestern Pacific

Jiasong Fang[1,2*], Chiaki Kato[3*], Gabriella M. Runko[2], Yuichi Nogi[3], Tomoyuki Hori[4], Jiangtao Li[5], Yuki Morono[6], and Fumio Inagaki[6,7,8]

[1]Hadal Science and Technology Research Center, Shanghai Ocean University,

Shanghai, China.

[2]Department of Natural Sciences, Hawaii Pacific University, Honolulu, Hawaii, U.S.A.

[3]Department of Marine Biodiversity Research, Japan Agency for Marine-Earth Science and Technology (JAMSTEC), Yokosuka, Japan.

[4]Environmental Management Research Institute, National Institute of Advanced Industrial Science and Technology (AIST), Ibaraki, Japan.

[5]State Key Laboratory of Marine Geology, Tongji University, Shanghai, China.

[6]Kochi Institute for Core Sample Research, JAMSTEC, Kochi, Japan.

[7]Research and Development Center for Ocean Drilling Science, JAMSTEC, Yokohama, Japan.

[8]Research and Development Center for Submarine Resources, JAMSTEC, Yokosuka, Japan.

1 Supplementary Figures and Tables

1.1 Supplementary Figures

Supplementary Figure 1. IODP Exp. 337 drill site C0020 (41°10.5983“N, 142°12.0328”E) off the coast of Shimokita Peninsula, Japan (from http://www.jamstec.go.jp/chikyu/exp337/e/findings.html).

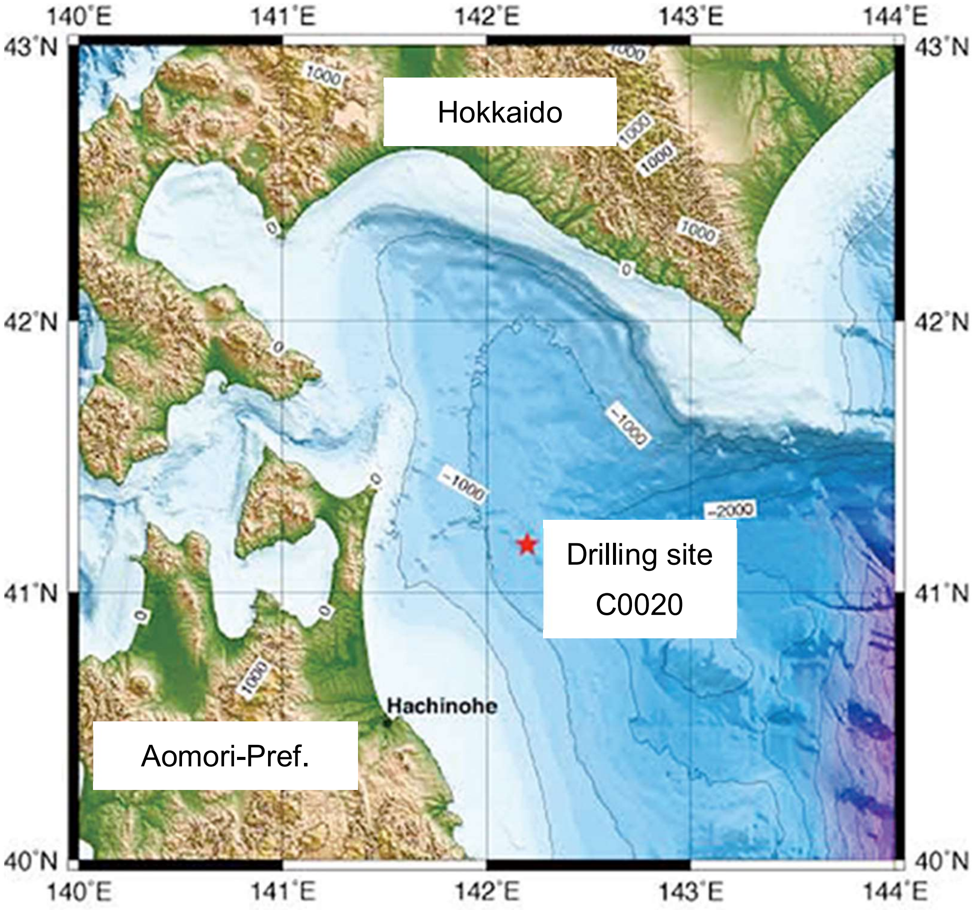

Supplementary Figure 2. Transmission electron micrographs of bacterial isolates 19R1-5 (a) and 29R7-12 (b), and scanning electron micrographs of the isolates 19R1-5 (c) and 29R7-12 (d).

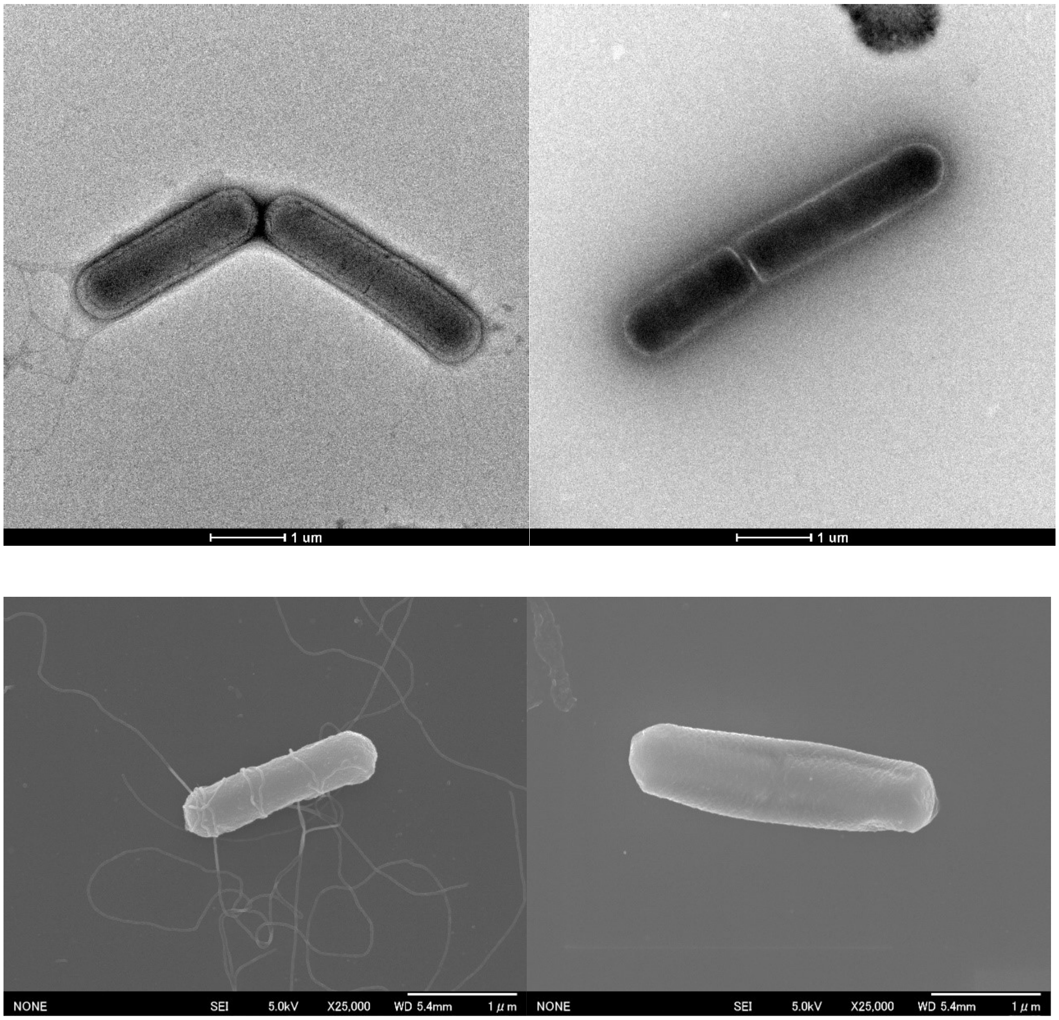

Figure S3. Relative abundance of bacteria at the phylum level (A), order (B) and genus level (C). Each color represents the percentage of the phylum (A) or order (B) or genus (C) in the total effective sequences of each sample.

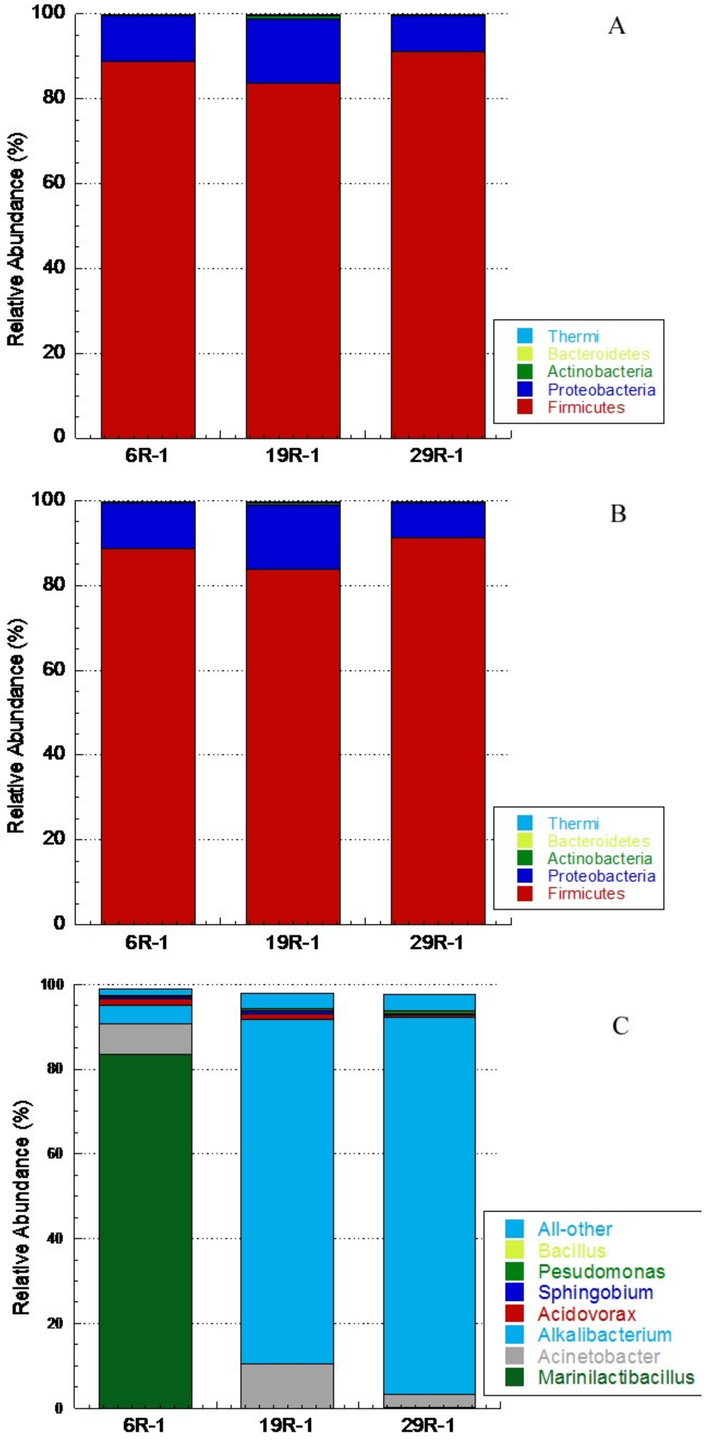

Supplementary Figure 4. Principal component analysis (PCoA) of bacterial 16S rRNA gene sequences obtained from different HP cultivation pools for sediments (6R-1, 19R-1, 29R-1) and medium aqueous phase (6R-2, 19R-2, 29R-2).

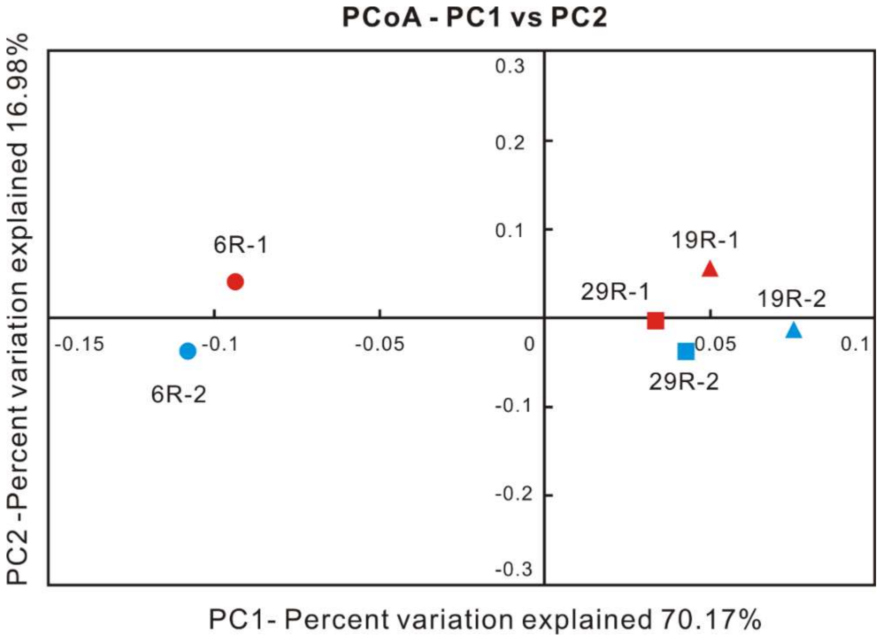

1.2 Supplementary Tables

Supplementary Table 1. G+C contents and levels of homology for the chromosomal DNAs of the subsurface isolates and the closely related species.

Strain	G+C content (mol%)	% DNA-DNA hybridization with:		
		6R3-1	19R1-15	*V. pantothenticus*
6R3-1	38.3	100	100	91.7
19R1-15	38.4	98.2	100	96.9
V. pantothenticus	38.0	100	100	100

Strain	G+C content (mol%)	% DNA-DNA hybridization with:		
		6R3-15	29R7-12	*B. subtillis*
6R3-15	45.1	100	100	90.7
29R7-12	44.8	94.9	100	75.8
B. subtillis	45.0	70.4	87.3	100

Supplementary Table 2. Comparison of physiological characteristics between the isolates and related species.

Substrate	*V. pantothenticus*	6R3-1	19R1-5	*B. subtilis*	6R3-15	29R7-12
Control	−	−	−	−	−	−
Glycerol	−	±	±	±	+	+
Erythritol	−	−	−	−	−	−
D-arabinose	−	−	−	−	−	−
L-arabinose	−	−	−	+	+	+
D-ribose	+	+	+	+	+	+
D-xylose	−	−	−	+	±	±
L-xylose	−	−	−	−	−	−
D-adonitol	−	−	−	−	−	−
Methyl-βD-xylopyranoside	−	−	−	−	−	−
D-galactose	+	+	+	−	−	−
D-glucose	−	+	+	+	+	+
D-fructose	−	±	+	+	+	+
D-mannose	+	+	+	+	+	+
L-sorbose	−	−	−	−	−	−
L-rhamnose	±	+	+	±	−	−
Dulcitol	−	−	−	−	−	−
Inositol	−	−	−	+	+	+
D-mannitol	−	−	−	+	+	+
D-sorbitol	−	+	+	+	+	+
Methyl-αD-mannopyranoside	−	−	−	−	−	−
Methyl-αD-glucopyranoside	−	±	+	−	±	−
N acetyl glucosamine	+	+	+	−	±	−
Amygdalin	+	+	+	±	−	−
Arbutin	+	±	+	±	−	−
Asculine ferric citrate	+	+	+	+	+	+
Salicin	+	+	+	±	−	−
D-cellobiose	−	+	+	+	−	−
D-maltose	+	+	+	+	+	+
D-lactose	−	−	−	−	−	−
D-melibiose	−	−	−	+	−	−
D-sucrose	+	+	+	+	+	+
D-trehalose	+	+	+	+	+	+
Inulin	−	−	−	−	+	+
D-melezitose	−	−	−	−	−	−
D-raffinose	−	−	−	−	−	−
Starch	+	±	+	±	−	−
Glycogen	−	−	−	+	−	−
Xylitol	−	−	−	−	−	−
Gentiobiose	−	+	+	−	−	−
D-turanose	+	±	+	+	−	−
Dlyxose	−	−	−	−	−	−
D-tagatose	+	+	+	−	−	−
D-fuctose	−	−	−	−	−	−
L-fuctose	±	+	+	−	−	−
D-arabitoL	−	−	−	−	−	−
L-arabitol	−	−	−	−	−	−
Gluconate	−	−	−	−	−	−
2 Keto gluconate	−	−	−	−	−	−
5 Keto gluconate	−	−	−	−	−	−
